# Effects of modified sediments on the growth of submerged macrophyte *Vallisneria natans* under low light conditions

**DOI:** 10.3389/fpls.2024.1450786

**Published:** 2024-09-05

**Authors:** Wenhao Xiong, Xiaowen Ma, Yonghong Xie, Wenwen Zeng

**Affiliations:** ^1^ Technology Innovation Center for Ecological Conservation and Restoration in Dongting Lake Basin, Ministry of Natural Resources, Changsha, China; ^2^ Key Laboratory of Agro-Ecological Processes in Subtropical Region, Institute of Subtropical Agriculture, Chinese Academy of Sciences, Changsha, Hunan, China; ^3^ Dongting Lake Station for Wetland Ecosystem Research, Institute of Subtropical Agriculture, Chinese Academy of Sciences, Changsha, Hunan, China; ^4^ University of Chinese Academy of Sciences, Beijing, China

**Keywords:** submerged plants, *Vallisneria natans*, modified sediment, low-light, vegetation reconstruction

## Abstract

Submerged plants are an important part of aquatic ecosystems, and the restoration of submerged plants is a key step in the reconstruction of aquatic ecosystems. However, little is known about the role of modified sediments in helping submerged plants recover under low light. In this study, we set up four sediment types and two light intensities to explore the effects of modified sediments on the growth of *Vallisneria natans* under two low light conditions. The results showed that the independent and interactive effects of light intensity and sediment type significantly affected the biomass, morphology, photosynthetic pigment content and antioxidant enzyme activity of *V. natans*. At 5% and 20% natural light intensity, the sediment modified with 40% peat soil had a larger root biomass and the highest leaf and root C/N ratio, the sediment modified with 40% vermiculite had a longer root length and more ramets. At 5% natural light intensity, the sediments modified with fly ash had shorter root length and smaller leaf biomass. The sediments modified with fly ash had the greatest chlorophyll content at 20% natural light intensity. It can be concluded that the addition of 40% peat soil or 40% vermiculite in sediment is conducive to the growth of *V. natans* under low light conditions. Our study indicates the positive effects of the modified sediment on the growth of *V. natans* under low light conditions, and our study will provide a reference for the restoration of submerged plants in aquatic ecosystems.

## Introduction

In recent years, with the development of industry and agriculture, people have discharged a large amount of pollutants into the water body, resulting in the extinction of aquatic plants and accelerating the degradation of the lake ecosystem (Abell et al., 2019). Submerged plants are producers of aquatic ecosystems, and they have an important impact on the structure and function of lake ecosystems (Dhote and Dixit, 2009; Li et al., 2020a). At present, the reconstruction of submerged plants in water bodies is an important part of the prevention and control of lake eutrophication, which is recognized by most scholars (Anda et al., 2016; Song et al., 2017; Wang et al., 2019a). After the recovery of submerged plants, the rate of nutrient cycling in the water body was reduced, and the overgrowth of phytoplankton was controlled. Therefore, the restoration of submerged plants is an important measure to rebuild a healthy lake ecosystem (Su et al., 2019).

However, in heavily polluted water bodies, it is difficult for submerged plants to survive and recover naturally (Zhu et al., 2016). As the basis for the survival of submerged plants, lake sediments provide various nutrients and inorganic elements for submerged plants, but excessive sediment fertility may also become an important factor limiting the growth of submerged plants (Xiao et al., 2007). After being affected by wave disturbances, the sediments will release pollutants and suspended particulate matter into the water body again (Wang et al., 2024). The contents of suspended particulate matter are the key factors affecting the underwater light environment (Dong et al., 2021). Photosynthesis is the most important metabolic activity of submerged plants, and the weakening of light intensity is the main factor limiting the growth and survival of submerged plants (Cao et al., 2019; Li et al., 2023a). Therefore, reducing the pollution load in sediments and improving the physicochemical conditions of sediments are the basis for the restoration of submerged plants (Wang et al., 2022).

Sediment restoration technologies primarily consist of sediment dredging, aeration, and chemical addition (Li et al., 2023b). However, these methods often require significant manpower and material resources, and they can also lead to secondary pollution (Zhong et al., 2008). Therefore, more and more attention has been paid to the *in-situ* improvement of sediments. Natural minerals such as bentonite (Liu et al., 2021), zeolite (Yang et al., 2014), and attapulgite (Yin and Kong, 2015) are common substrate improvement materials, and environmental friendliness is a common feature of these materials (Bai et al., 2022). Materials such as attapulgite and calcite remodel the bacterial community structure of sediments under iron-modified conditions and can affect the microbial driven phosphorus cycling in sediments (Jin et al., 2023). Bentonite and calcium peroxide modified with lanthanum can reduce soluble iron in sediment pore water and enhance internal phosphorus removal from sediments (Chen et al., 2024). The addition of biochar to sediments promotes the growth of submerged plants (Li et al., 2020b), while lanthanum-modified attapulgite combined with *Vallisneria natans* can be used to fix phosphorus in various sediments. The addition of these materials to the sediment contributes to ecological restoration (Kong et al., 2024).

Vermiculite is a product formed after high-temperature calcination of natural minerals, with a special layered structure, strong adsorption capacity and cation exchange capacity (Rama et al., 2019). Peat soil is rich in organic matter, which can promote plant growth and inhibit the migration of heavy metals in the soil (Kumpiene et al., 2007; Wang et al., 2019b). Fly ash is a product of burning pulverized coal in power station to generate electricity, which can inhibit the uptake of arsenic and mercury by plants (Sánchez et al., 2024). However, most of these materials are used in the study of soil amendments, but their use as sediment amendments to aid in the recovery of aquatic organisms is less well known.

*V. natans* is a common submerged plant in freshwater ecosystems, and it is often used for wetland and shallow lake vegetation restoration due to its strong pollutant absorption capacity and tolerance to low-light conditions. Due to its strong fruiting ability, strong reproductive ability, and wide distribution, *V. natans* often dominates the local aquatic ecosystem (Lin et al., 2020). Therefore, *V. natans* was chosen for this experiment. In this study, we set up four sediment types and two light intensities to explore the effects of modified sediments on the growth of *V. natans* under two low light conditions, in order to provide a scientific basis for the ecological restoration of degraded aquatic ecosystems.

## Materials and methods

### Experimental design

The experiment was conducted from September 17 to November 16, 2023 at the Datong Lake Sub-station of Dongting Lake Wetland Ecosystem Research Station (N 29°12′7″, E 112°33′28″). The sediment was collected from Datong Lake to remove shells and larger impurities and bring it back to the laboratory. The improved materials were peat soil, fly ash and vermiculite, and the addition amount was 40% of the total volume. The total nitrogen, total phosphorus and carbon content of the sediment were determined after modification (**Table 1**).

**Table 1:** Total nitrogen, total phosphorus and carbon content of the modified sediments.

Indicator	Group	Mean ± SD
TN(mg/kg)	Lake sediment (L)	1340 ± 69.28
	Lake sediment + 40% Peat soil (P)	1640 ± 121.24
	Lake sediment + 40% Fly ash (F)	1113.33 ± 32.15
	Lake sediment + 40% Vermiculite (V)	1456.67 ± 20.82
TP(mg/kg)	Lake sediment (L)	695 ± 52.27
	Lake sediment + 40% Peat soil (P)	652.33 ± 18.62
	Lake sediment + 40% Fly ash (F)	878.5 ± 46.59
	Lake sediment + 40% Vermiculite (V)	768.67 ± 26.57
C(%)	Lake sediment (L)	2.41 ± 0.31
	Lake sediment + 40% Peat soil (P)	2.79 ± 0.2
	Lake sediment + 40% Fly ash (F)	3.9 ± 0.01
	Lake sediment + 40% Vermiculite (V)	2.16 ± 0.02

**Figure 1:**
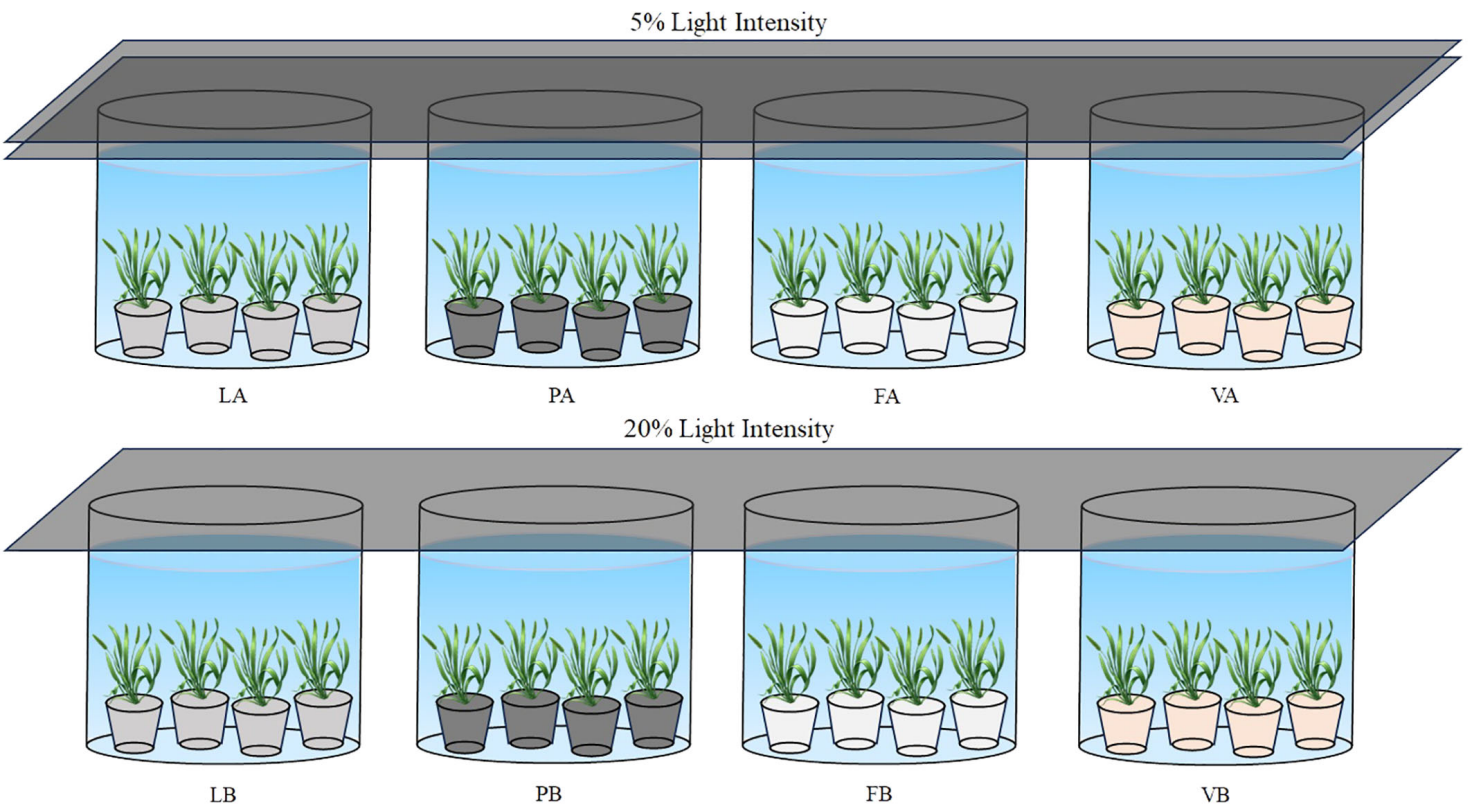
Schematic diagram of the experimental design. LA, Lake sediment + 5%Natural light; LB, Lake sediment + 20%Natural light; PA, Lake sediment + 5%Natural light + 40% Peat soil; PB, Lake sediment + 20%Natural light + 40% Peat soil; FA, Lake sediment + 5%Natural light + 40% Fly ash; FB, Lake sediment + 20%Natural light + 40% Fly ash; VA, Lake sediment + 5%Natural light + 40% Vermiculite; VB, Lake sediment + 20%Natural light + 40% Vermiculite.

The *V. natans* used in the experiment was purchased from a local ecological company. In order to ensure the uniformity of the study, we carefully selected healthy, intact, uniformly sized plants with no offspring ramets for the experiment. The *V. natans* were transplanted into a black bucket (opening diameter 20 cm, bottom diameter 18 cm, height 23 cm), filled with 15 cm of modified sediment, and then the black bucket was placed in a white bucket (diameter 110 cm, height 70 cm). The water depth was controlled at 60 cm and the light intensity was controlled using a black shade net so that the experiment was carried out under 5% natural light and 20% natural light. Additionally, a transparent plastic canopy was erected above the experimental setup to prevent rainwater ingress while allowing sunlight penetration. There were eight treatment groups in the experiment (**Figure 1**). Twenty replicates were set up for each treatment group, and the experiment was carried out for a total of 60 days. Four replicates of *V. natans* were randomly collected from each treatment group every 12 days for analysis of morphological and physiological indicators.

### Determination of plant indicators

After each harvest, the first step was to wash the impurities from the surface of the plant with water. Then, we used toilet paper to remove excess water and proceeded to measure the plant height and maximum root length, as well as record the number of clonal ramets. Additionally, we weighed 0.1g of plant leaves and soaked them in absolute ethanol for 48 hours in a lightless environment to extract chlorophyll. After extraction, we measured the absorbance at 645nm and 663nm wavelengths and used absolute ethanol as a blank to calculate chlorophyll a (Chl a) and chlorophyll b (Chl b) (Arnon, 1949).

And then, 0.5g of fresh plant leaves were placed in a pre-cooled mortar, 1mL of pre-cooled phosphate buffer was added to the mortar, 1mL of buffer was added after grinding in an ice bath, the slurry was poured into a centrifugal test tube, the mortar was washed with 2mL of buffer, and the remaining liquid was also poured into the centrifuge tube. After all samples were grinded, centrifuged at low temperature (0-4°C) for 20min (3000rpm), and the supernatant was collected for the determination of the activities of superoxide dismutase (SOD), peroxidase (POD) (Chen et al., 2014) and catalase (CAT) (Asghari et al., 2013). Root vigor is determined by measuring the amount of 2,3,5-triphenyltetrazolium chloride (TTC) (Prajitha and Thoppil, 2017) present in the roots.

Finally, the leaves and roots of the plant were dried to a constant mass (60°C, 48 h) and weighed for the determination of leaf biomass, root biomass. The root/leaf ratio is calculated as the ratio of root biomass to leaf biomass (Ma et al., 2021; Yang et al., 2022). The samples of plants were ground into powder and filtered through a 200-mesh sieve, and the carbon and nitrogen content of plant leaves and roots, as well as sediments, was determined by an elemental analyzer (Various MAX cube, Elementar, Germany), and the C/N ratio of plant leaves and roots were also calculated. Due to the small plant mass, several replicates of each treatment were mixed to dry and grind, and three parallel samples were determined. Sediments total phosphorus (TP) content was determined by the HClO_4_-H_2_SO_4_ digestion-molybdenum antimony anti colorimetric method (Parkinson and Allen, 1975).

### Statistical analysis

SPSS 27.0 software was used for data analysis, and three-way analysis of variance (ANOVA) was used to test the interactive and independent effects of time (T), light intensity (L) and sediment type (S) on the morphology, reproductive strategy and physiological characteristics of *V. natans*. The LSD method was used to compare the differences between different treatments. Before ANOVA, the data that do not satisfy the normal distribution and homogeneity of variance are transformed by square root, Ln and Box-Cox. Pearson correlation analysis was used to correlate the indicators of *V. natans*. The data images were plotted using Origin2022 software.

## Results

### Changes in biomass and reproductive strategies indicators of *V. natans*

**Figure 2:**
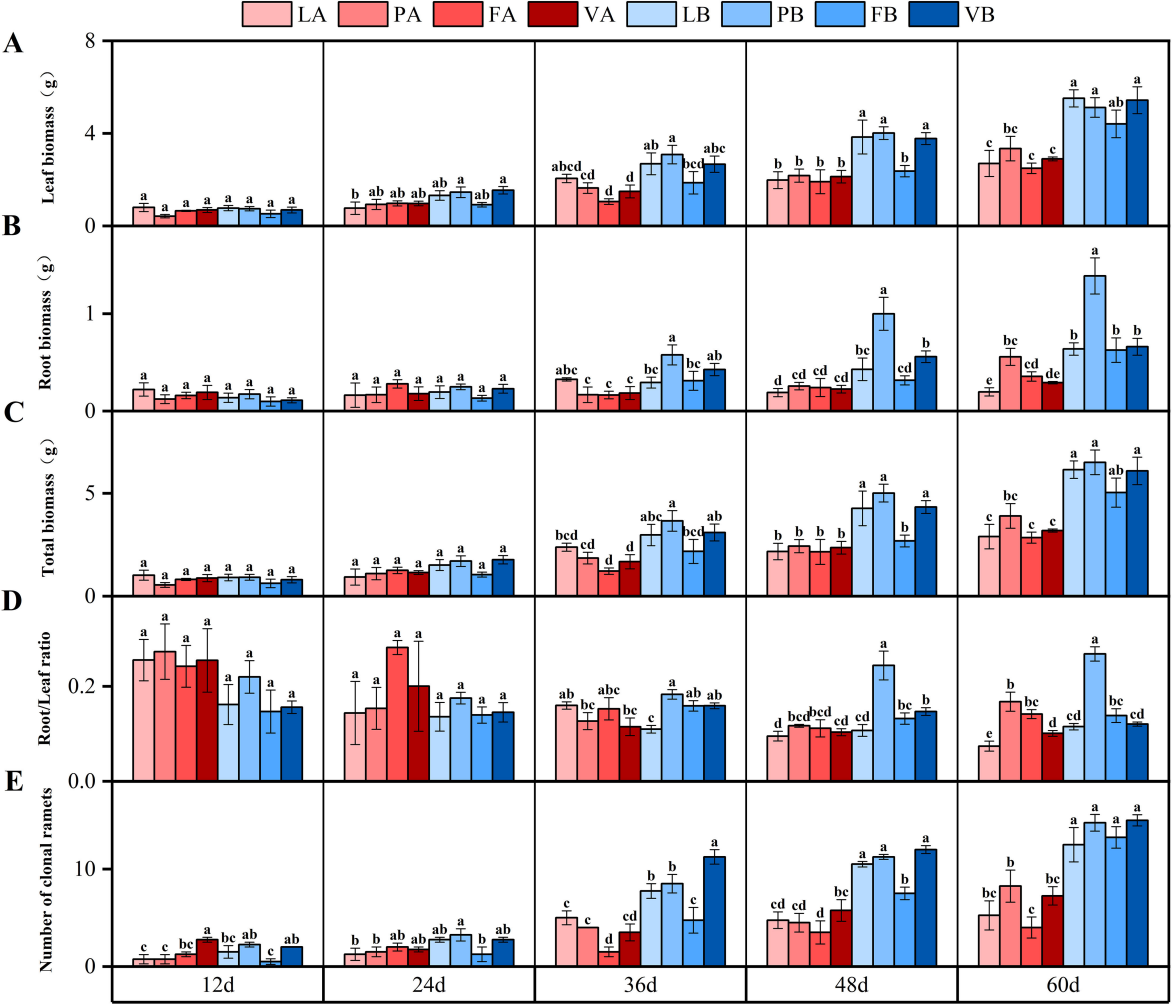
Changes in leaf biomass **(A)**, root biomass **(B)**, total biomass **(C)**, root/leaf ratio **(D)** and number of clonalramets **(E)** in different treatment groups during the experiment. Different letters represent significant differences (*P*<0.05).

The biomass of *V. natans* under different treatments increased with time. At 60 days, there was a significant difference in leaf biomass under different light treatments, and the leaf biomass of 20% light treatment was 79.1% higher than that of 5% light treatment (**Figure 2A**). The interaction of time and light intensity had a significant effect on leaf biomass (**Table 2**). There was a significant difference in root biomass under 20% light treatment at 48 days (*P*<0.05), the root biomass of the treatment with peat soil was 133.2% higher than that of the control group, and at the 60th day (**Figure 2B**). The interaction of light intensity and sediment had a significant effect on root biomass (**Table 2**). At 60 days, there was a significant difference in the total biomass under different light treatments (*P*<0.05), and the total biomass of 20% light treatment was 85.4% higher than that of 5% light treatment (**Figure 2C**). The interaction of time and light intensity had a significant effect on total biomass (**Table 2**).

**Table 2:** F-value and significance of three-way analysis of variance (ANOVA) results for the effects of Time (T), Light intensity (L) and Sediment type (S) on measures of biomass and reproductive strategies of *V. natans*.

	T		L		S		T*L		T*S		L*S		T*L*S	
	*F*	*P*	*F*	*P*	*F*	*P*	*F*	*P*	*F*	*P*	*F*	*P*	*F*	*P*
Total biomass	119.473	**<0.001**	99.361	**<0.001**	7.187	**<0.001**	15.880	**<0.001**	0.964	0.487	2.665	0.051	0.569	0.864
Leaf biomass	132.941	**<0.001**	100.402	**<0.001**	6.874	**<0.001**	14.437	**<0.001**	0.763	0.687	1.959	0.124	0.637	0.807
Root biomass	42.458	**<0.001**	64.998	**<0.001**	15.557	**<0.001**	17.203	**<0.001**	4.502	**<0.001**	10.614	**<0.001**	1.360	0.195
Root/leaf ratio	9.019	**<0.001**	0.284	0.595	6.132	**<0.001**	6.992	**<0.001**	1.384	0.183	4.080	**0.008**	0.606	0.833
Number of clonal ramets	162.720	**<0.001**	223.948	**<0.001**	17.509	**<0.001**	31.687	**<0.001**	1.956	**0.034**	1.551	0.205	1.624	0.094

Significant *P*-values are presented in bold.

With the increase of experimental time, the root/leaf ratio of the four groups under 5% light treatment showed a decreasing trend. At day 60, the root/leaf ratio of peat soil was significantly higher than that of the control group under the same light conditions (**Figure 2D**). The interaction of time and light intensity had a significant effect on root/leaf ratio, the interaction of light intensity and sediment had a significant effect on root/leaf ratio (**Table 2**).

On the 12th day, the number of clonal ramets increased faster with the 20% light treatment, and at the 60th day, there was a significant difference in the number of clonal ramets with different light treatments (*P*<0.05), and the number of clonal ramets with 20% light treatment was 114.5% higher than that under 5% light treatment (**Figure 2E**).

### Changes in morphological indicators of *V. natans*

**Figure 3:**
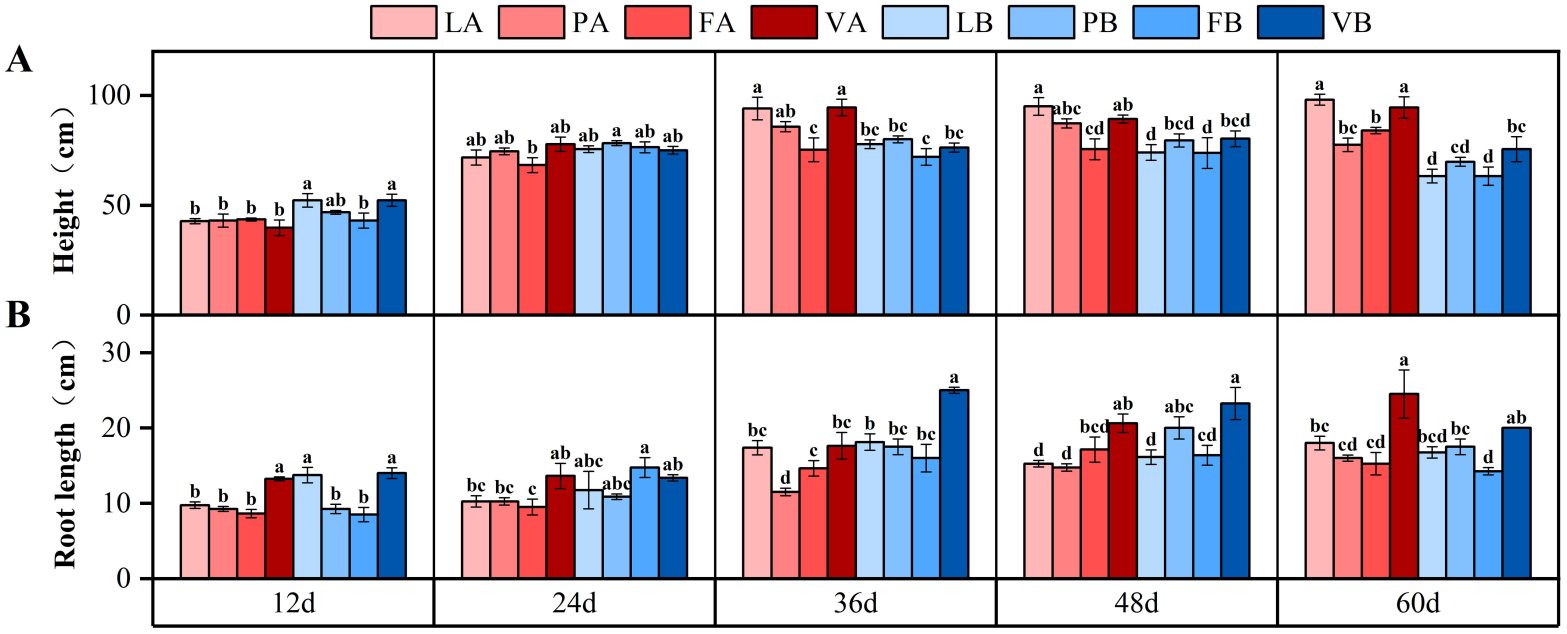
Changes in plant height **(A)** and root length **(B)** in different treatment groups during the experiment. Different letters represent significant differences (*P*<0.05).

The plant height and root length under different treatments increased with time. At the beginning of the experiment, the 20% light treatment had a higher plant height than the 5% light treatment on day 12. But with increasing time, the plant height growth rate of the 5% light treatment was greater than that of the 20% light treatment. On the 60th day, the plant height of 5% light treatment was 32.8% and 25.2% higher than that of 20% light treatment, respectively (**Figure 3A**). The interaction of time and light intensity had a significant effect on plant height (*P*<0.001), the interaction of light intensity and sediment had a significant effect on plant height, and the interaction of time, light intensity and sediment had a significant effect on plant height (**Table 3**).

**Table 3:** F-value and significance of three-way analysis of variance (ANOVA) results for the effects of Time (T), Light intensity (L) and Sediment type (S) on measures of morphology and physiology *V. natans*.

	T		L		S		T*L		T*S		L*S		T*L*S	
	*F*	*P*	*F*	*P*	*F*	*P*	*F*	*P*	*F*	*P*	*F*	*P*	*F*	*P*
Height	173.551	**<0.001**	37.012	**<0.001**	11.565	**<0.001**	22.297	**<0.001**	1.435	0.159	3.851	**0.011**	2.443	**0.007**
Root length	68.942	**<0.001**	15.778	**<0.001**	38.418	**<0.001**	4.908	**0.001**	2.399	**0.008**	1.098	0.353	2.634	**0.004**
Chl(a+b)	0.723	0.578	188.631	**<0.001**	18.230	**<0.001**	25.266	**<0.001**	2.957	**0.001**	9.884	**<0.001**	1.660	0.084
Chla	0.578	0.679	179.435	**<0.001**	19.352	**<0.001**	28.857	**<0.001**	3.364	**<0.001**	10.340	**<0.001**	1.861	**0.046**
Chlb	11.785	**<0.001**	184.213	**<0.001**	14.030	**<0.001**	16.867	**<0.001**	2.363	**0.009**	7.853	**<0.001**	1.402	0.174
Leaf C/N	10218.9	**<0.001**	28722.6	**<0.001**	8391.3	**<0.001**	4955	**<0.001**	2754.2	**<0.001**	5575.9	**<0.001**	1355	**<0.001**
Root C/N	327.3	**<0.001**	262.6	**<0.001**	947.5	**<0.001**	160.5	**<0.001**	186.2	**<0.001**	283.8	**<0.001**	28.4	**<0.001**
TTC	0.958	0.434	0.757	0.386	5.529	**0.001**	1.542	0.208	2.027	**0.028**	2.408	0.071	0.951	0.485
SOD	85.166	**<0.001**	52.793	**<0.001**	2.648	0.052	11.563	**<0.001**	2.265	**0.013**	0.936	0.426	1.885	**0.043**
POD	47.687	**<0.001**	29.215	**<0.001**	4.830	**0.003**	2.271	0.066	1.723	0.070	1.189	0.317	0.837	0.612
CAT	0.772	0.545	4.849	**0.030**	3.060	**0.031**	1.821	0.129	1.078	0.384	0.587	0.625	1.593	0.102

Significant *P*-values are presented in bold.

On the 60th day, the root length of vermiculite treatment was the largest under 5% light, and there was a significant difference between vermiculite treatment and control group (*P*<0.05), which was 36.1% higher than that of control group (**Figure 3B**). The interaction of time, light intensity and sediment had a significant effect on root length (**Table 3**).

### Changes in chlorophyll indicators of *V. natans*

**Figure 4:**
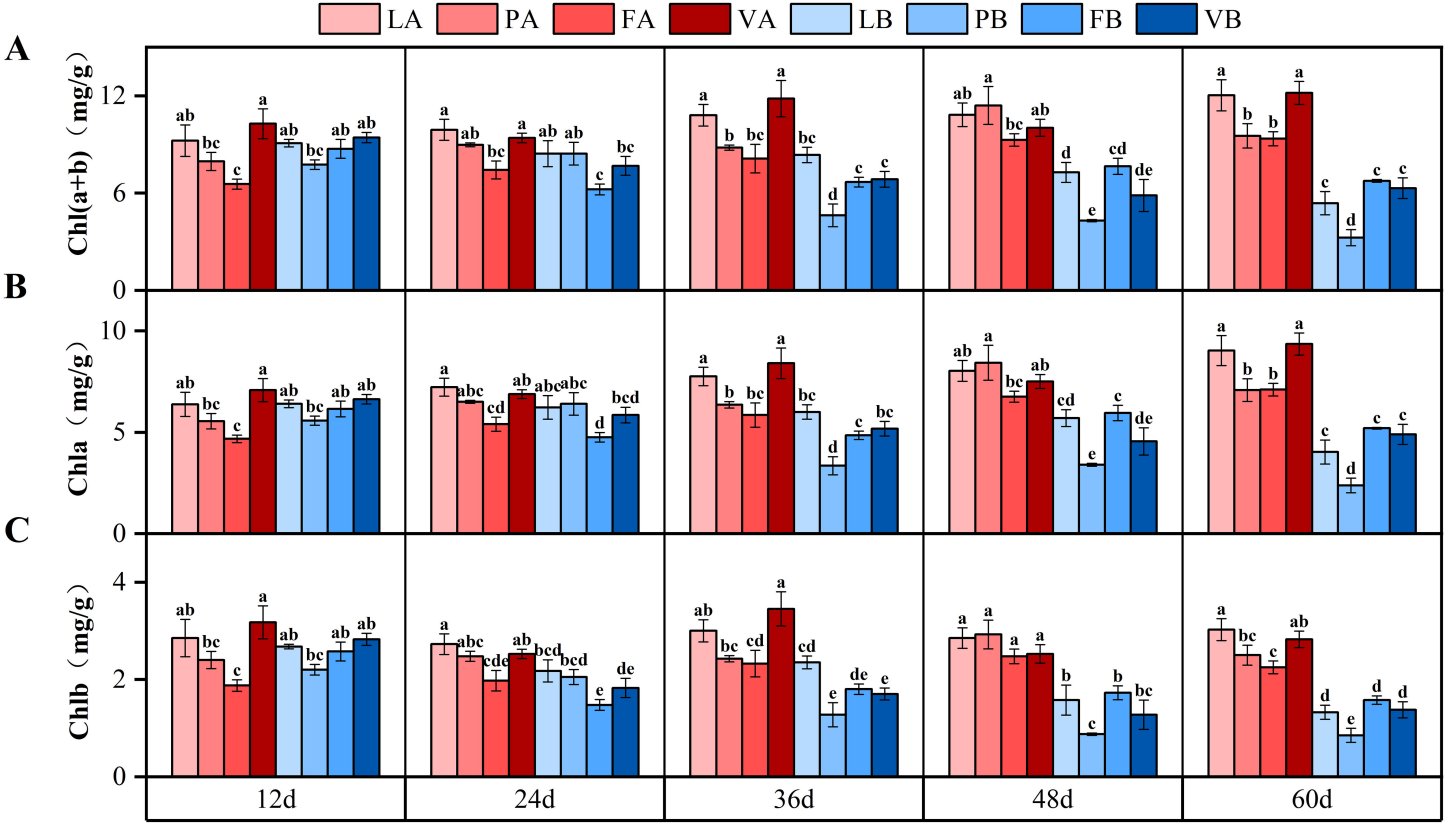
Changes in Chl(a+b) **(A)**, Chla **(B)** and Chlb **(C)** in different treatment groups during the experiment. Different letters represent significant differences (*P*<0.05).

The chlorophyll (a+b) of different light treatments had different trends, the 5% light treatment had an increasing trend, which increased by 26.7% on the 60th day compared with the 12th day, while the 20% light treatment had a decreasing trend, and the 60th day decreased by 38% compared with the 12th day. At day 60, there was a significant difference in chlorophyll (a+b) between different light treatments (*P*<0.05), and chlorophyll (a+b) was 98.7% higher in 5% light treatment than in 20% light treatment. The trend of chlorophyll a and chlorophyll b was similar to that of chlorophyll (a+b) (**Figures 4A–C**). The interaction of light intensity and sediment had a significant effect on chlorophyll indexes, and the interaction of time, light intensity and sediment had a significant effect on chlorophyll a (**Table 3**).

### Changes in C/N ratio and root vigor of *V. natans*

**Figure 5:**
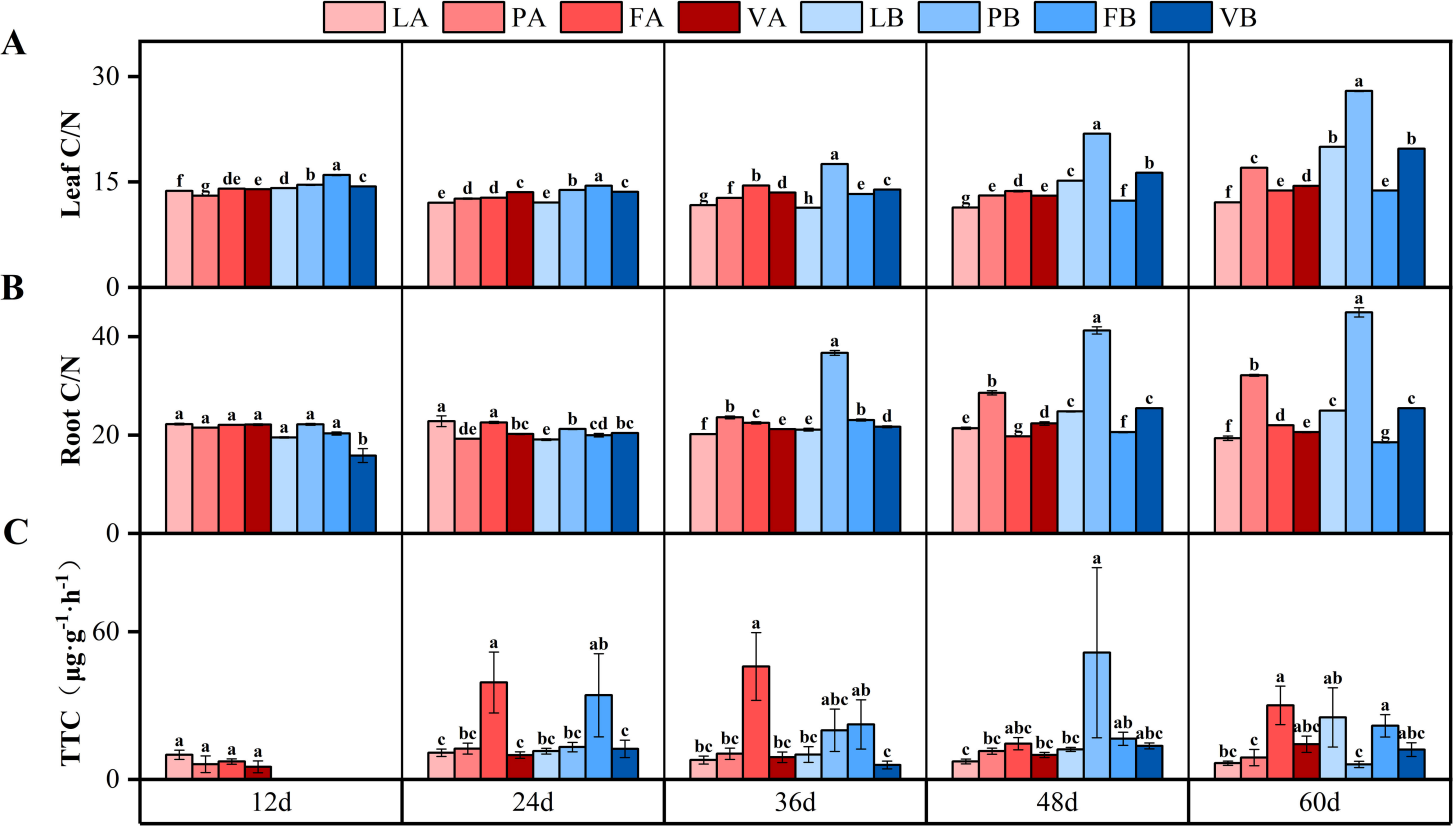
Changes in leaf C/N ratio **(A)**, root C/N ratio **(B)** and root vigor (TTC) **(C)** in different treatment groups during the experiment. Different letters represent significant differences (*P*<0.05).

The C/N ratio of leaves in the peat soil treatment group under 20% light showed an upward trend, the C/N ratio of leaves in the 20% light treatment group was 39.8% higher than that of the control group at 60 days. The C/N ratio of leaves in the fly ash treatment group under 20% light showed a decreasing trend, the C/N ratio of leaves in the fly ash treatment group under 20% light was 31.7% lower than that of the control group at 60 days and there was a significant difference (**Figure 5A**). The interaction of light intensity and sediment type had a significant effect on the C/N ratio of leaves and roots (**Table 3**).

The C/N ratio of roots in the peat soil treatment group showed an upward trend. On the 36th day, the C/N ratio of roots in the peat soil treatment group was significantly different from that of the control group under 5% and 20% light, and increased to the maximum value on the 60th day, which was 66% and 80% higher than that of the control group, respectively (**Figure 5B**). The interaction of light intensity and sediment type had a significant effect on the C/N ratio of roots (**Table 3**).

The root vigor of the fly ash treatment group under 5% light was always at a high level and was significantly different from that of the control group (*P*<0.05), while the root vigor of the peat soil and fly ash treatment group under 20% light was more unstable (**Figure 5C**). The interaction of time and sediment type had a significant effect on root vigor (**Table 3**).

### Changes in physiological indexes of *V. natans*

**Figure 6:**
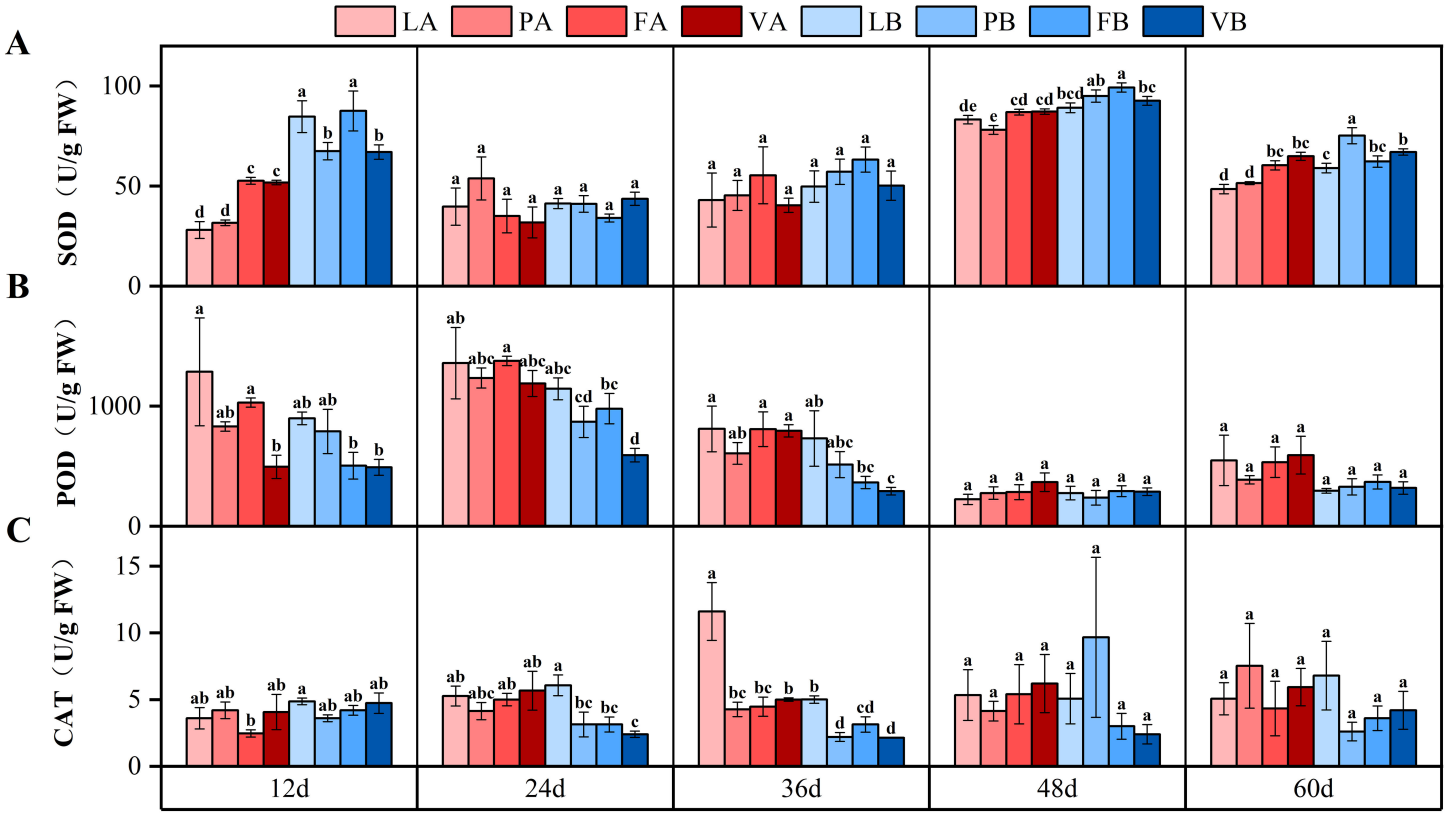
Changes in superoxide dismutase (SOD) **(A)**, peroxidase (POD) **(B)** and catalase (CAT) **(C)** contents of *V. natans* leaves in different treatment groups. Different letters represent significant differences (*P*<0.05).

On the 24th day after the start of the experiment, the SOD content in plant leaves in all treatment groups began to increase, and the SOD content of plant leaves in 20% light treatment was 24.1% higher than that in 5% light treatment (**Figure 6A**). The interaction of time, light intensity and sediment type had a significant effect on SOD (**Table 3**).

Contrary to the trend of SOD in plant leaves, from the 24th day of the experiment, the POD content in plant leaves in all treatment groups had a decreasing trend, and the POD content in plant leaves treated with 20% light treatment was 29.7% lower than that in 5% light treatment (**Figure 6B**).

There was no obvious change trend of CAT content in plant leaves between different treatment groups, and the CAT content in plant leaves treated with 5% light treatment was 26.7% higher than that in 20% light treatment during the whole experiment, and the difference in CAT content between groups was not significant and statistically significant (**Figure 6C**).

### Pearson correlation analysis of indicators of *V. natans*

**Figure 7:**
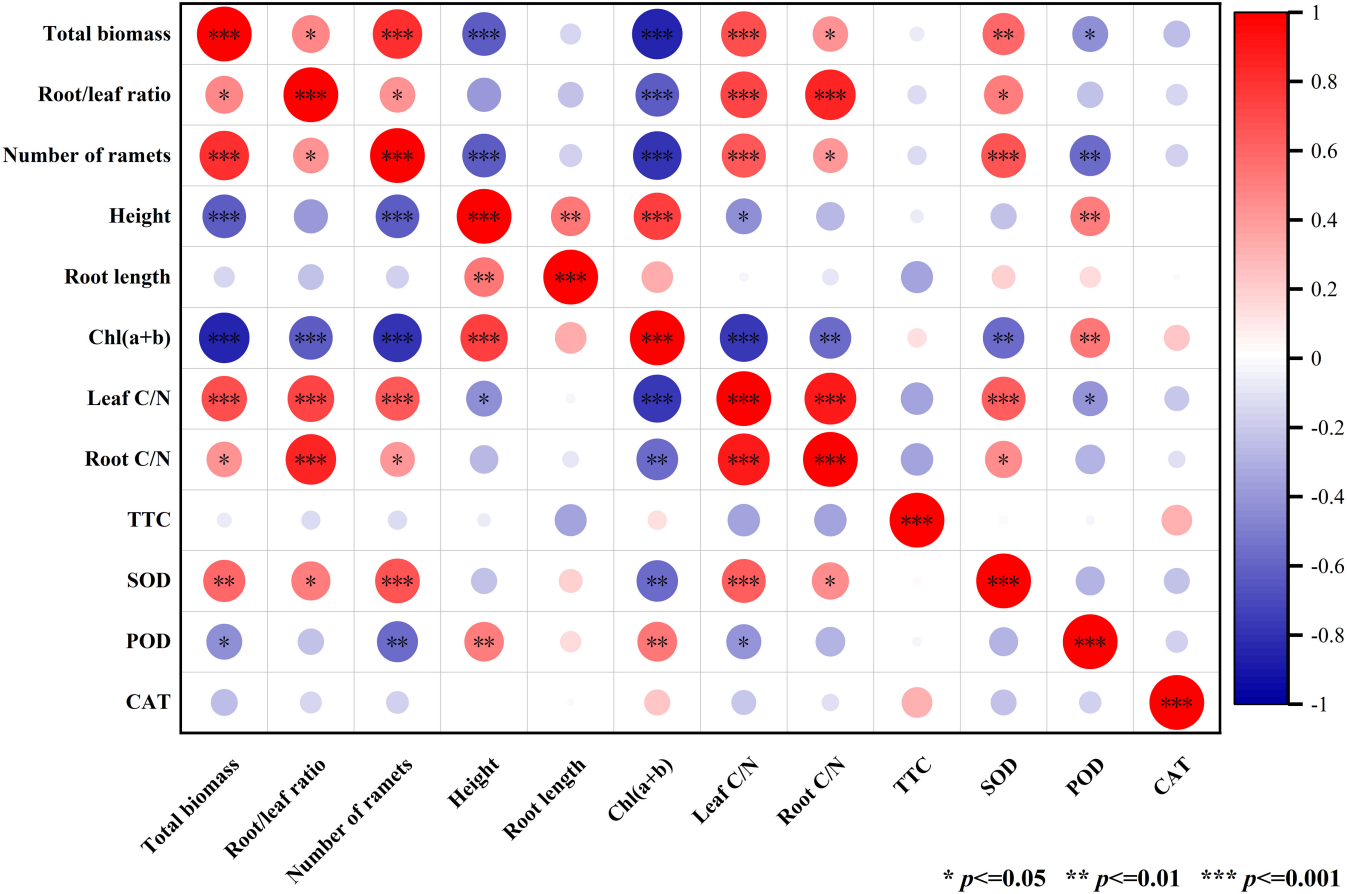
Pearson correlation analysis of indicators of *V. natans*.

The biomass of *V. natans* had a significant positive correlation with number of clonal ramets, leaf C/N ratio and SOD, while the biomass of *V. natans* had a significant negative correlation with chlorophyll (a+b) and plant height (**Figure 7**). Plant height was significantly positively correlated with root length and chlorophyll (a+b). The content of SOD was significantly positively correlated with the number of clonal ramets and leaf C/N (**Figure 7**).

## Discussion

In this experiment, *V. natans* showed a series of responses to the reduction of light intensity, including changes in biomass, number of clonal ramets, plant height, and chlorophyll. And these response differences gradually increased between different treatment groups over time. *V. natans* is a common submerged plant in the middle and lower reaches of the Yangtze River, it has a strong adaptability to environmental changes, its growth strategy often changes with the change of environment (Zhang et al., 2020), it has a rosette-type base, disperses in the sediment through oblique stolons and spreads horizontally above the ground (Xiao et al., 2006), so it has a strong tolerance to low light (Yu et al., 2016). When the light intensity does not reach the compensation point for plant growth, submerged plants adapt to changes in the environment by changing their growth patterns, such as increasing plant height, chlorophyll and decreasing fresh weight (Chou et al., 2022). As the experiment time increases and the light intensity decreases from 20% to 5%, the root biomass, leaf biomass and ramets of *V. natans* decreased, while the plant height and chlorophyll increased, so that more light energy could be obtained under low light conditions. Submerged plants can absorb most of the mineral nutrients from the sediment through their roots. Studies have shown that fertile sediments stimulate plant growth (Zhu et al., 2012), but too much nutrients in sediments can also have direct or indirect negative effects on the growth of submerged plants (Xu et al., 2016). In this experiment, the root biomass and root length of *V. natans* were different among different sediment types, with greater root biomass and root/leaf ratio in the peat soil treatment group, smaller root length in the fly ash treatment group, and larger root length in the vermiculite treatment group. Some studies have found that the reason for the decrease in root length may be that the nutrient concentration of the sediment is sufficient for the growth of submerged plants and does not require too much root development of plants (Wang et al., 2023a). And the increase in root length is due to the addition of vermiculite that makes the sediment loose, thereby promoting the root growth of submerged plants (Wang et al., 2023b).

The difference in chlorophyll content between different light treatment groups gradually increased with the increase of experimental duration. Chlorophyll is the most important pigment for photosynthesis of submerged plants (Yan et al., 2023), the content of chlorophyll in plant leaves reflects the photosynthetic capacity and growth status of plants, the main function of chlorophyll a is to convert light energy into electrical energy for electron transfer, and finally into chemical energy, chlorophyll b is the main component of plant photosynthetic pigment system (Anderson and Aro, 1994). In this experiment, when the light intensity was reduced from 20% to 5%, both chlorophyll a and chlorophyll b increased significantly, and the chlorophyll a and chlorophyll b in the 5% light treatment group were 97.6% and 107.7% higher than those in the 20% light treatment group, respectively, indicating that *V. natans* responded to low light stress by increasing the content of photosynthetic pigments and the efficiency of photosynthesis. Under the same light intensity, the chlorophyll content of the peat soil treatment group was lower, combined with the high root/leaf ratio of this treatment group, it can be inferred that the lack of essential substances for chlorophyll synthesis in the sediment makes the plants prioritize the root development to obtain sufficient nutrients.

Due to the addition of different amendments to the sediment, some of the corresponding indicators of *V. natans* also changed with the addition of different amendments. Nitrogen is one of the most important elements for plant growth, and insufficient or excessive nitrogen will affect the synthesis of photosynthetic pigments and the imbalance of carbon and nitrogen metabolism, thus further limiting the growth of plants (Shi et al., 2020). Under the combined effect of low light and high nutrients, submerged plants may have relevant reactions such as changes in carbon and nitrogen ratio and oxidative stress (Cronin and Lodge, 2003; Zhang et al., 2010). In this experiment, the difference between leaf C/N ratio and root C/N ratio was small under different light treatments, while the difference between leaf C/N ratio and root C/N ratio was large under different sediment treatments. And the difference between the peat soil treatment group and the control group gradually increased with the increase of time. The results showed that although the total nitrogen content in the peat soil treatment group was the highest, the sediment in the peat soil treatment group lacked nitrogen due to the non-decomposition of nitrogen-containing substances, which led to the restriction of plant growth, resulting in the phenomenon of large plant root biomass and yellowing of leaves in the peat soil treatment group. Root vigor is an important indicator to reflect the root status of submerged plants, which can not only represent the growth status of plant roots, but also reflect the stress resistance of plants (Liu et al., 2020). In this experiment, different light intensities did not have a great effect on root vigor, while the root vigor of plants treated with different sediment types was quite different. At 20% light intensity, the root vigor of the fly ash treatment was very high, and there were significant differences between the control group on day 24 and day 36, and then slowly decreased, indicating that the plants may be slowly adapting to the sediment environment brought by fly ash. The root vigor of the treatment group using vermiculite was at a low level under the two types of light, indicating that vermiculite could provide a more suitable sediment environment for the root development of *V. natans*. Through this experiment, it can be inferred from the correlation of *V. natans* indicators that the content of carbon and nitrogen in sediments may affect the C/N ratio of plant tissues, thereby changing the biomass of plants under low light, so that plants can better adapt to low light environment.

The three antioxidant enzymes (Zhou et al., 2019), SOD, POD and CAT, play a key role in plant growth (Zhu et al., 2020), and changes in antioxidant enzymes can also reflect whether plants are subject to growth stress (Li et al., 2022). On the 12th day of this experiment, the SOD activity of the 5% light treatment group was significantly lower than that of the 20% light treatment group, and there was no significant difference in the SOD activity of the two light treatments with the increase of experimental time, indicating that at the beginning of the experiment, the 20% light treatment group was under greater stress, but with the increase of time, different treatment groups adapted to the environment. In this experiment, the POD activity of 5% light treatment was higher than that of 20% light treatment, but the difference was not significant, and the POD activity of all treatment groups decreased with the increase of experimental time, indicating that different treatment groups were gradually able to adapt to the new environment during the growth process. The CAT content of the fly ash treatment group was always at a low level. The difference between different light treatment groups was not significant, which was consistent with the changes of SOD activity and POD activity, indicating that 5% light intensity and 20% light intensity had little effect on the antioxidant enzyme activity of *V. natans*. Time, as an important factor in plant growth, plays an irreplaceable role, and experiments in different periods or durations will have different results. In this experiment, the changes in the various indicators of the *V. natans* were mainly due to the change of time.

## Conclusion

The growth status and physiological characteristics of *V. natans* were significantly affected by the independent and comprehensive effects of time, light intensity, and sediment type. As light intensity decreases, *V. natans* adapted to environmental changes by adjusting its morphological and physiological characteristics, such as reducing the number of clonal ramets, decreasing the root/leaf ratio, increasing the photosynthetic pigment content and plant height. Different sediment types also affected the morphology and physiology of *V. natans*. Under low light intensity and extremely low light intensity, the sediment modified with peat soil had a larger root biomass and a higher leaf and root C/N ratio, the sediment modified with vermiculite had a longer root length and more ramets. Under extremely low light intensity, the sediments modified with fly ash had shorter root length and smaller leaf biomass. Under low light intensity, the sediments modified with fly ash had greater chlorophyll content. This study demonstrates that incorporating peat soil, fly ash, and vermiculite into sediment under low light stress alters its physical and chemical properties. These amendments potentially facilitate the growth of the submerged plant *V. natans*. Therefore, this study further clarified the role of modified sediments in ecological restoration projects, which is of great significance for the restoration of submerged plants and good management of aquatic ecosystems.

## Data Availability

The raw data supporting the conclusions of this article will be made available by the authors, without undue reservation.
